# Genetic Analyses of a Three Generation Family Segregating Hirschsprung Disease and Iris Heterochromia

**DOI:** 10.1371/journal.pone.0066631

**Published:** 2013-06-26

**Authors:** Long Cui, Emily Hoi-Man Wong, Guo Cheng, Manoel Firmato de Almeida, Man-Ting So, Pak-Chung Sham, Stacey S. Cherny, Paul Kwong-Hang Tam, Maria-Mercè Garcia-Barceló

**Affiliations:** 1 Department of Surgery, Li Ka Shing Faculty of Medicine, The University of Hong Kong, Hong Kong SAR, China; 2 Department of Psychiatry, Li Ka Shing Faculty of Medicine, The University of Hong Kong, Hong Kong SAR, China; 3 Center for Genomic Sciences, Li Ka Shing Faculty of Medicine, The University of Hong Kong, Hong Kong SAR, China; 4 Centre for Reproduction, Development, and Growth, Li Ka Shing Faculty of Medicine, The University of Hong Kong, Hong Kong SAR, China; 5 State Key Laboratory of Brain and Cognitive Sciences, The University of Hong Kong, Hong Kong SAR, China; 6 Faculdade de Medicina de Ciências Medicas de Minas Gerais, Belo Horizonte, Brazil; Ohio State University Medical Center, United States of America

## Abstract

We present the genetic analyses conducted on a three-generation family (14 individuals) with three members affected with isolated-Hirschsprung disease (HSCR) and one with HSCR and heterochromia iridum (syndromic-HSCR), a phenotype reminiscent of Waardenburg-Shah syndrome (WS4). WS4 is characterized by pigmentary abnormalities of the skin, eyes and/or hair, sensorineural deafness and HSCR. None of the members had sensorineural deafness. The family was screened for copy number variations (CNVs) using Illumina-HumanOmni2.5-Beadchip and for coding sequence mutations in WS4 genes (*EDN3*, *EDNRB*, or *SOX10*) and in the main HSCR gene (*RET*). Confocal microscopy and immunoblotting were used to assess the functional impact of the mutations. A heterozygous A/G transition in *EDNRB* was identified in 4 affected and 3 unaffected individuals. While in EDNRB isoforms 1 and 2 (cellular receptor) the transition results in the abolishment of translation initiation (M1V), in isoform 3 (only in the cytosol) the replacement occurs at Met91 (M91V) and is predicted benign. Another heterozygous transition (c.-248G/A; -predicted to affect translation efficiency-) in the 5′-untranslated region of *EDN3* (EDNRB ligand) was detected in all affected individuals but not in healthy carriers of the *EDNRB* mutation. Also, a *de novo* CNVs encompassing *DACH1* was identified in the patient with heterochromia iridum and HSCR

Since the *EDNRB* and *EDN3* variants only coexist in affected individuals, HSCR could be due to the joint effect of mutations in genes of the same pathway. Iris heterochromia could be due to an independent genetic event and would account for the additional phenotype within the family.

## Background

Waardenburg-Shah syndrome, Waardenburg-Hirschsprung disease or Type IV Waardenburg syndrome (WS4; MIM #277580) is a congenital developmental disorder characterized by pigmentary abnormalities of the skin, eyes and/or hair, sensorineural deafness and aganglionosis of variable portions of the colon (Hirschsprung disease) [1]. WS4 is rare, with an incidence of ∼1/40,000–1/50,000 live-births, and occurs in all races. To the best of our knowledge, there are only 74 WS4 cases described (2012) in the English literature [2], [3], [4], [5], [6], [7], [8]. WS4 can occur sporadically or it segregates in families in an autosomal dominant or recessive inheritance pattern with reduced penetrance [8], [9].

WS4 is underlain by homozygous or heterozygous coding sequence (CDS) mutations affecting any of the three following genes, *EDN3* (20q13.2–q13.3), encoding the endothelin-3 peptide; *EDNRB* (13q22), encoding its receptor; and SRY (Sex determining region Y)-box 10 (*SOX10*) (22q13.1), encoding a transcription factor [8], [10]. Chromosomal abnormalities involving the regions where these genes map have also been described. These genes regulate the proliferation, migration and differentiation of the neural crest cells (NCCs) from which melanocytes, enteric and peripheral neurons derive. In human, *SOX10* CDS mutations account for about 50% of the WS4 patients and CDS mutations in *EDN3* or *EDNRB* for 20–30% of the WS4 patients [8]. However, 15–35% of the WS4 patients are devoid of CDS mutations in these 3 genes suggesting that mutations either in regulatory regions or CDSs of other genes may also lead to this disorder. Recently, a *de novo* deletion encompassing *SOX10* regulatory elements have been identified in a WS4 patient with no CDS mutations, underscoring the relevance of regulatory sequences in rare disorders [11]. In addition, the intra- or inter- familial variability of the phenotype and incomplete penetrance observed in patients with mutations in the known WS4 genes, also suggest the involvement of additional unknown loci that may modify the effect of the mutations in the main WS4 genes.

Importantly, heterozygous mutations in *EDNRB* or *EDN3* are also known to cause isolated HSCR [12], [13]. Moreover, genetic interaction between *EDNRB* and *RET*, (the main HSCR gene) [14], [15] has been described in a kindred segregating both, isolated HSCR and WS4 [16] as well as in families or sporadic patients with isolated-HSCR [17], [18], [19]. The fact that two mutated genes are needed for the manifestation of the disease also implies functional interaction, with additional molecules or “mediators” mediating the link between the RET and EDNRB signaling pathways [20]. Interactions between *Sox10*, *Edn3* and *Ednrb* during enteric nervous system and melanocyte development have also been described [21] in mice. Indeed, the complexity and phenotypic variability already observed in genetically heterogeneous or oligogenic congenital disorders can be conceptually understood in the light large range of molecular and cellular interactions that take place during development. Thus, DNA alterations in any of the genes codifying for the signaling molecules that govern the fate of the melanocytes and enteric neurons precursors may represent a primary etiology for WS4. The WS4 phenotype may therefore result from i) single severe DNA alterations in coding or non-coding sequences of any genes encoding crucial molecules; ii) the combinatory effects of less severe mutations in several genes; iii) the former and the later combined. The existence of non-clinically affected individuals carrying mutations in major genes invokes a compensatory effect by other genes.

Here we present candidate gene screening, haplotype and copy number variation analyses on a three-generation family segregating Hirschsprung disease and WS4 features. We observed that while heterozygous mutations in two genes of the same pathway only coexisted in HSCR-affected individuals, a *de novo* CNV could account for the heterochromia iridum phenotype presented by the only family member displaying WS4 features.

## Materials and Methods

### Pedigree

**Figure 1:**
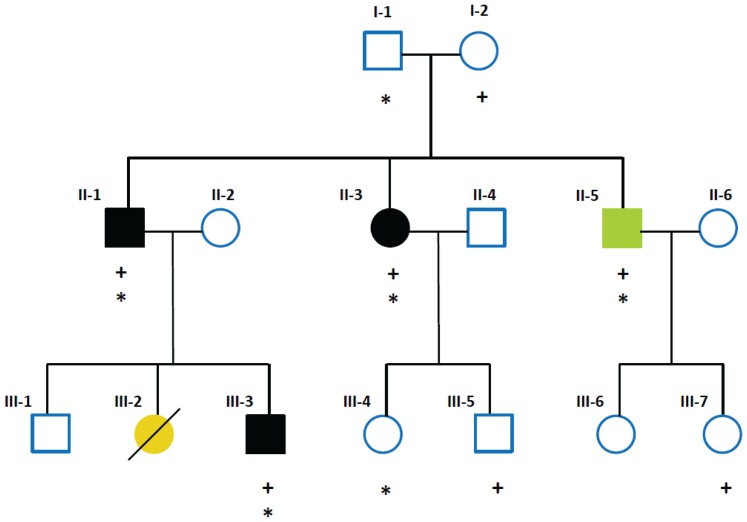
Pedigree of the three-generation family included in this study. Slash: deceased. Solid black: individuals affected with HSCR only. Green: individual affected with HSCR and heterochromia iridum. Yellow: individual affected with HSCR and meningocele. + indicates *EDNRB* c.1A>G; * indicates *EDN3* c.-248G>A.

A non-consanguineous three-generation white Brazilian family of European ancestry (14 individuals) with Hirschsprung phenotype was included in this study. Out of the fourteen individuals, three (II-1, II-3, III-3) were only affected with the most severe type of Hirschsprung disease (long segment aganglionosis; L-HSCR) and one (II-5) was affected with L-HSCR and complete heterochromia iridum (**Figure 1**). Adult family members gave written informed consent and for minors, written informed consent was obtained from their parents. The study was approved by the institutional review board of The University of Hong Kong together with the Hospital Authority (IRB:UW06-349/T/1374).

**II-1** was born in 1965 with Hirschsprung disease from unaffected non-consanguineous parents. Duhamel procedure was performed in 1966, when he was 17 months old. Complications following the operation included stenosis and fistulas and, an emergency colostomy was performed. Finally, after a Soave operation, the patient was discharged (in 1968) in excellent conditions. Colonic biopsy showed aganglionosis. **II3**, born in 1968, was medically treated until the age of 3, when she was submitted to a Soave operation (without colostomy) and finally diagnosed with L-HSCR. **II5** was born (1970) with complete heterochromia iridum (the 2 irides are of different color, one blue and the other brown) and L-HSCR. He was treated medically till he was 3 years old when he was also submitted to a Soave procedure without colostomy. Colonic biopsy showed aganglionosis. **III-3** born in 2007 with L-HSCR, was operated at the age of 15 months. Colonic biopsy showed aganglionosis. III-3 has two siblings, **III-1**, a male born in 1994 with no HSCR or WS4 features, and **III-2**, a girl (born in 2004) who died aged 10 months. III-2 was born with myelomeningocele and megacolon. She developed post operatory mild hydrocephalus and passed away to septicaemia and urinary infection. No DNA sample is available from this family member. This is the only family member who had neurological problems.

Except for **II5**, no pigmentation anomalies were observed in any of the family members. Also, none of the individuals had sensorineural hearing impairment nor developmental/growth delay or dysmorphic features.

### DNA sequence analysis

#### Direct sequencing of coding regions

Polymerase chain reaction (PCR) amplification and direct sequencing were used to screen for DNA variations in the coding and the 5′ and 3′ flanking regions of the known WS4 candidate genes *EDN3*, *EDNRB*, *SOX10*, as well as those of *RET* (the main HSCR gene). For the latter, the *RET* intron 1 SNP (rs2435357), highly associated with HSCR and known to interact with *EDNRB* [18] was also amplified and sequenced. Primers and PCR conditions are described elsewhere [22], [23], [24].

#### SNP genotyping array and CNV predictions

The 14 individuals were genotyped on the Illumina HumanOmni2.5-Quad Beadchip, which contains 2,443,177 markers throughout the genome. This allowed us to **i)** verify the familial relationships; **ii)** assess the presence of CNVs; **iii)** obtain accurate haplotypes.

Calls were provided by the Centre for Applied Genomics (TCAG; www.tcag.ca; Toronto, Canada). CNV segments were predicted by two programs, PennCNV [25] and QuantiSNP [26], which are two most efficient and publicly available CNV calling algorithms for Illumina data [27]. Both programs implement hidden Markov models based on normalized probe intensity value against expected intensity in terms of Log_2_ R ratio and relative allelic copy in terms of B allele frequency. PennCNV integrates additional information in CNV prediction (i.e. population allele frequency and distance between adjacent SNPs). .

To obtain high-confidence calls, we only used the overlapping region of CNVs called by both programs. Before selecting the overlapping CNV regions, quality control were done separately (please refer to **supplementary materials for details**).

### Haplotype reconstruction

The most likely haplotypes of the pedigree members were constructed using MERLIN [28]. The MERLIN haplotype output files were visualized with HaploPainter [29].

### Generation of expression constructs

Wild-type *EDNRB* variant 1 cDNA (RefSeq: NM_000115.1) cloned in a pCMV6 expression vector (pCMV6-*EDNRB*-v1-GFP) with a variant of green fluorescent protein tag at the C-terminal end (Turbo-GFP; Origene) and an empty pCMV6-GFP expression vector were obtained from Origene Technologies (Rockville, MD, USA). Reverse transcription PCR (RT-PCR) was performed to isolate full-length coding region of *EDNRB* variant 4 (RefSeq: NM_001201397; isoform 3) from human melanoma cell lines and ligated into pGEM-T Easy vector (Promega). Primers with Sgf I and Mlu I restriction sites were designed to amplify full-length coding regions of *EDNRB* variant 4 (RefSeq: NM_001201397) which was then subcloned to pCMV6 entry vector to generate a pCMV6-*EDNRB*-v4-GFP vector. The *EDNRB* mutations vectors pCMV6-*EDNRB*-v1-M1V-GFP (M1V for isoforms 1) and pCMV6-*EDNRB*-v4-M91V-GFP (M91V for isoform 3) were generated by site-directed mutagenesis using the QuickChange XL Site-Directed Mutagenesis Kit (Stratagene) according to the manufacturer's instructions. All the DNA fragments were verified by direct sequencing. Primer sequences are available upon request.

### Cell lines and Transfection

Human Embryonic Kidney 293 cells (HEK293, Invitrogen) were grown in Dulbecco's modified Eagle medium (DMEM) supplemented with 10% fetal calf serum (FBS), penicillin (100 U/ml) and streptomycin (100 µg/ml) at 37°C with the presence of 5% CO_2_. Cells were seeded in culture wells 24 hours prior to transfection. The plasmids, pCMV6-AC-GFP (empty vector), pCMV6-AC-*EDNRB*-v1-GFP (wild-type EDNRB isoform 1), pCMV6-AC-*EDNRB*-v4-GFP (wild-type EDNRB isoform 3), pCMV6-AC-*EDNRB*-v1-M1V-GFP (M1V) and pCMV6-AC-*EDNRB*-v4-M91V-GFP (M91V), were transfected into HEK293 cells according to Lipofectamine® 2000 transfection reagent (Invitrogen) instructions.

### Immunofluorescence

HEK293 cells (2×10^5^), grown for 24 h in each 35 mm Petri-dish with 14 mm Bottom Microwell No. 1.5 coverglass (MatTek, USA), were transfected with 1.5 µg empty vector, wild-type EDNRB isoform 1, wild-type EDNRB isoform 3, M1V and M91V using Lipofectamine® 2000 transfection reagent (Invitrogen). The living cells were incubated overnight and GFP fluorescence were observed directly on the chamber slide under confocal laser scanning using a Zeiss LSM 700 confocal laser-scanning microscope (λ_ex_ = 488 nm,λ_em_>515 nm). Fluorescence intensity was measured using the eight-bit gray scale of the LSM 700 software.

## Results and Discussion

### Pedigree: pattern of inheritance

We considered the mode of inheritance or genetic mechanism that might give this pattern of affected family members. Two unaffected parents (I-1, I-2) had three affected children (II-1, II-3, II-5), one of whom (II-1) passed down the phenotype to two individuals (III-2, III-3) of the third generation in an **apparently autosomal dominant manner**. If that was the case, it would imply that one of the parents carried a mutation that, for whatever reason, was not fully penetrant. Other possibilities considered were **i)** one of the parents carried a **balanced translocation** that became unbalanced in the children in which case, miscarriages and multiple malformations in multiple organs would have been expected; and **ii)** **autosomal recessive** inheritance, in which case, **I-1**, **I-2** and **II-2** would all have to be carriers for the same autosomal recessive disease, which is extremely unlikely as this is a non-consanguineous family. Yet, the transmission of WS4 is described as neither fully recessive nor fully dominant. WS4 patients with different pathogenic mutations in the same gene (compound heterozygotes) have also been described [8].

Importantly, while only II-5 (heterochromia iridum) and III-2 (neurological problems) have with characteristics of WS4, the rest of the affected family members manifest isolated HSCR.

After all these considerations, we set out to investigate the genetic lesions underlying this variable phenotype. To this end, and, to cover all possibilities, the 14 family members were screened for small variations in the coding regions of **i)** the WS4 candidate genes; **ii)** the main HSCR gene; and **iii)** CNVs through the genome as deletions encompassing the candidate genes have been reported and translocations could also be considered in spite of all of the above.

### No*SOX10* deleterious variants

Sequencing of the complete CDS of the *SOX10* gene did not reveal any damaging variant that could have explained the phenotype. Two SNPs leading to synonymous changes were identified in non-affected members of the family (Table 1). These SNPs, rs73415876 (p.Y83Y) and rs139884 (p.H309H) have a global Minor Allele Frequency of 0.033 and 0.287 respectively.

**Table 1:** Sequence variants of*EDNRB*, *EDN3*, *RET* and *SOX10* identified.

Gene and exon	Nucleotide change (s)	Codon position and amino acid change	Individuals
*EDNRB*			
Exon 1	c.1A/G	M1V	I-2, **II-1, II-3**, *II-5*, **III-3**, III-5, III-7
Exon 6	c.831A/G (rs5351)	L277L	I-1, **II-1**, **II-3**, II-4, II-6, III-1, **III-3**, III-4, III-5, III-6, III-7
*EDN3*			
5′UTR	c.-248G/A		I-1,**II-1**, **II-3**, *II-5*, **III-3**, III-4
*RET*			
Exon 2	c.135G/A (rs1800858)	A45A	I-1, I-2, **II-1**, II-2, **II-3**, *II-5*, II-6, III-1, **III-3**, III-4, III-5, III-6, III-7
Exon 7	c.1296A/G (rs1800860)	A432A	I-2, II-2, **II-3**, *II-5*, II-4, II-6, III-1, **III-3**, III-4, III-5, III-6, III-7
Exon 13	c.2307T/G (rs1800861)	L769L	I-1, I-2, **II-1**, **II-3**, II-4, *II-5*, III-1, **III-3**, III-4, III-5, III-6, III-7
*SOX10*			
Exon 2	c.249C>T (rs73415876)	Y83Y	II-6
Exon 4	c.927T>C (rs139884)	H309H	II-6, II-4, III-6, III-7

In bold: HSCR affected individuals; In italics: individual affected with WS4.

### Identification of a heterozygous mutation in*EDNRB*

Sequencing of the complete CDS of the *EDNRB* gene revealed a c.1A>G heterozygous DNA change in the NM_000115.3 sequence. The DNA variant was not only found in all affected individuals (II-1, II-3, II-5, III-3) but was also present in three unaffected individuals (I-2, III-5, III-7) (**Table 1** **;** **Figure 1**). c.1A>G was passed down to the family members by the unaffected mother (I-2). This variant was not present in any of the databases searched (NCBI, dbSNP137, 1000 Genomes Project –release 13, December 2012-). This variant resulted in the replacement of the translation initiation codon methionine (Met) with a valine (Val) in the EDNRB isoforms 1 and 2 (NP_000106.1 and NP_003982.1; 442 and 436 amino acids, respectively) and such replacement (M1V) would theoretically abolish the use of the translation initiation codon (ATG-GTG, M1V). However, in EDNRB isoform 3 (NP_001188326.1; 532 amino acids) the replacement is at Met91 (M91V) and is predicted benign by PolyPhen [30].

At this point, the only explanation we could provide is that of reduced pentrance as direct sequencing of the other WS4 candidate genes or *RET* did not reveal any pathological mutation in their CDSs. Yet, we formulated several hypothesis to account for the intra-familial phenotypic variability: **i)** since different *EDNRB* transcripts are expressed concomitantly (at least in the still developing newborn's gut [31] the phenotype manifestation could depend on the relative expression amounts of the mutated EDNRB isoforms (benign/damaging) at a given time point during the embryonic stages, provided that all isoforms play a similar role on neural crest cell development –enteric neurons/skin melanocytes-; **ii)** in carriers, in spite of having the initiation codon (M1V) mutated in isoform 1 and 2, the protein is translated using an alternative translation start site and the resulting protein, although shorter, still is functional [32]; **iii)** while the amount of protein expressed from the wild-type *EDNRB* allele is sufficient to maintain proper signalling in healthy carriers, it does not reach the necessary threshold for signalling in affected individuals; and/or **iv)** differential allelic expression (or imprinting) between carriers and affected individuals exists. Thus, variance in expression levels at different time points could account for the intra-familial variability, including that of individual II-5. Yet, no matter which of the mechanism hypothesized is proved or disproved, there must be additional genetic factors to account for the reduced penetrance of this mutation and the intra-familial variability of the phenotype. For obvious reasons, these postulations cannot be fully tested. Nevertheless, we set out to explore some of the possibilities mentioned with regards to the EDNRB mutation.

#### The M1V mutation abolishes the EDNRB isoform 1

To investigate the translocation of the mutant EDNRB proteins within cells, we transiently transfected equal amounts (20 µg) of the wild-type or M1V mutant GFP-tagged *EDNRB* isoform 1 into HEK293 cells and analysed by confocal microscopy.

**Figure 2:**
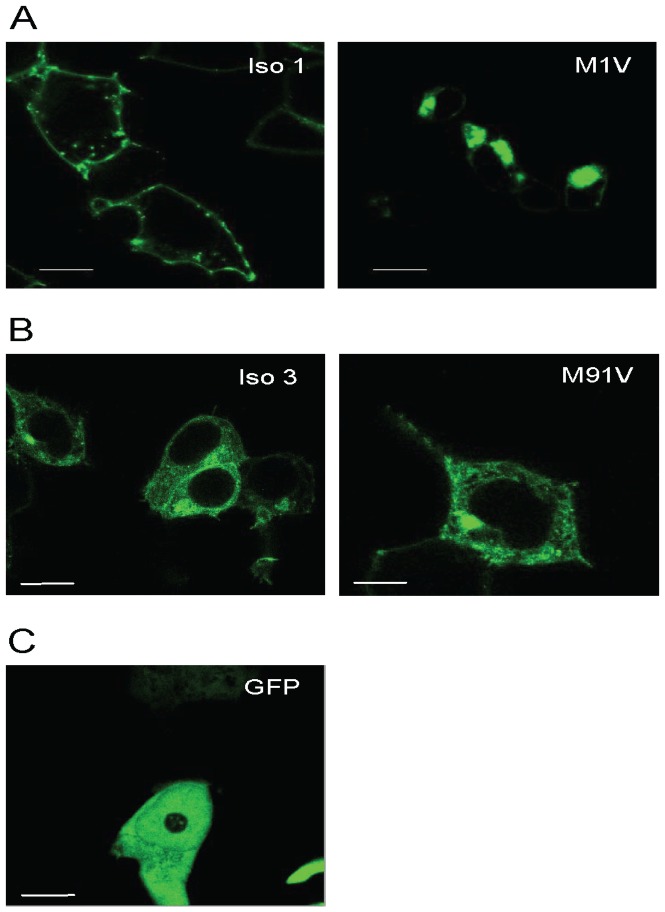
Localization of C-terminally GFP-tagged EDNRB fusion proteins in HEK293 cells. A: HEK293 cells were transiently transfected with wild-type EDNRB isoform 1 (Iso 1) or mutant EDNRB isoform 1 (M1V). Signals were observed at the plasma membrane in cells expressing wild-type isform 1 while signals were detected in the cytosol in cells transfected with mutant isoform 1. B: HEK293 cells were transiently transfected with wild-type EDNRB isoform 3 (Iso 3) or mutant EDNRB isoform 3 (M91V). Signals were found in the cytosol in cells transfected with either wild-type or mutant isoform 3. C. HEK293 cells were transiently transfected with GFP vector only. Shown are the representative iamges. Bars, 50 µm.

In cells transfected with wild-type EDNRB isoform 1/GFP, signals were detected at the plasma membrane only (Figure 2A). This indicates that EDNRB isoform 1 protein translocated to the cell surface and fulfilled its functional property as a cell surface receptor. On the other hand, the mutant EDNRB isoform 1 (M1V) displayed a clustered localization in the cytosol and a significantly reduced intensity on the membrane when compared with the wild-type (**Figure 2A**). Our interpretation is that the mutation (M1V) abolishes the translation start site and that the mutated RNA could have been translated through an alternative translation initiation codon. Sequence inspection revealed that the alternative translation initiation codon is likely to be located between Gly26 and Arg64, where, within a sequence compatible with the Kozak consensus, there is a methionine in codon 46 (Met46). Thus, a shorter protein, that could not translocate onto the membrane due to the lack of the signal peptide, could have been generated and account for our observations. Reduced amount of EDNRB receptor might alter the signalling pathway and hence, the differentiation and development of neural crest cells could be affected.

#### Role of the*EDNRB* isoform 3

We then investigated whether indeed the mutation was innocuous to EDNRB isoform 3 as predicted by PolyPhen. Confocal microscopy showed that both wild-type EDNRB isoform 3 and mutant EDNRB isoform 3 (M91V) were imperceptible on the plasma membrane (Figure 2B), which implies that this isoform may not function as a cellular receptor. We then resorted to TargetP 1.1 [33] and MitoProt II v1.101 [34] for *in silico* prediction of the EDNRB isoform 3 subcellular location (Table S1). TargetP assigns the location in accordance with the predicted presence of any of the N-terminal presequences including mitochondrial targeting peptide (mTP) and secretory pathway signal peptide (SP) and MitoProt is designed to identify mitochondrial signal sequences. Both programs predicted the presence of a 115 residues long mTP in the N terminal of EDNRB isoform 3 (Figure S1). Moreover, according to MitoProt, the probability of being exported to mitochondria hardly differed between the wild-type and mutated EDNRB isoform3 (92.41% and 89.60% respectively). Importantly, no mTP were predicted for EDNRB isoforms 1 or 2. Therefore, it would appear that M91V would not affect the putative role of the EDNRB isoform 3 in the cytoplasma organelle. Thus, the EDNRB isoform 3 might not play a role as a cellular receptor and, consequently, nor in the EDNRB signaling controlling the neural crest cells fate.

While we cannot demonstrate whether variation in expression levels of EDNRB isoform 1 (mutated or while type) during development played a role in the manifestation of the phenotype, it seems that postulations **i)** and **ii)** are to be dismissed. Still, the effect of more than one gene is patent by the transmission and variability of the phenotype in this family.

### No interaction between*RET* and *EDNRB* alleles in this family

Given the significant association between the transmission of the *EDNRB* Trp276Cys mutation and an HSCR-susceptibility *RET* haplotype reported in HSCR-trios [18] as well as the finding of two functionally significant *EDNRB* and *RET* mutations in a patient with isolated HSCR [19], we investigated the CDS of the *RET* gene and the HSCR-risk haplotype in all 14 family members.

No *RET* CDS pathological variants or mutations were detected in these individuals. Next, to find out whether a link could be established between the presence of the *RET* risk haplotype and the *EDNRB* mutation with regards to the penetrance of the latter, we constructed 4 markers *RET* haplotypes and investigated their transmission within the family (**Figure S2**). The *RET* risk haplotype consists of the HSCR-associated alleles “TAGG” of rs2435357; rs1800858, rs1800860, and rs1800861 polymorphisms. Importantly, it has been shown that rs2435357T allele (*RET* intron 1) as well as the *RET* risk haplotype are linked to reduced *RET* expression both *in vitro* and *in vivo* [35], [36], [37]. Therefore, the joint presence of the *EDNRB* mutation and the *RET* risk haplotype in affected individuals could account for the phenotypic differences between healthy and affected *EDNRB* mutation carriers. Yet, we found that the *RET* risk haplotype (both in homo- and heterozygosis states) was indistinctly present in affected and non-affected carriers as well as in non-carriers (**Figure S2**). Therefore, it seems that the phenotypic variability cannot be explained by the interaction of these two *RET* and *EDNRB* alleles.

### Joint effect of the*EDNRB* and *EDN3* alleles

As no CDS pathogenic variants had been found in *EDN3*, we paid attention to a G to A change (c.-248G/A) in the 5′ untranslated region (5′UTR) of the gene. Importantly, all **affected individuals carrying the** ***EDNRB*** **mutation** (II-1, II-3, II-5 and III-3; **Figure 1**) also had this *EDN3* variant. The c.-248G/A *EDN3* change was not present in healthy ***EDNRB*** carriers (III5, III7) but present in two healthy non-carriers, one of whom, I-1 (unaffected father) passed it down. Haplotype reconstruction (**Figure** S**3**) clearly showed the transmission of the -248G/A *EDN3* variant in the pedigree from the unaffected father (I-1). This *EDN3* variant is not reported in dbSNP137, or in the 1000 Genomes Project database (release 13, December 2012).

As post-transcriptional regulation is mediated by *cis*-acting regulatory motifs in the 5′ and 3′UTRs, we used bioinformatic tools to find out if the c-248G/A overlapped with any of such motifs and whether the replacement of a G with an A would be detrimental. Importantly, the -248G/A transition occurred in a highly evolutionary conserved site (**Figure S4**). Such degree of conservation for a non-translated base suggests that this 5′UTR guanine may be critical for the binding of regulatory proteins, and any disruption of this process would destabilise the mRNA and/or affect translation efficiency. While according to ENCODE , this position resides in the binding site of transcription factor SUZ12, JASPAR, a transcription factor binding profile database http://jaspar.genereg.net/ did not identify any on the site itself, but several in the neighbourhood. Thus, the variant might reduce access to those neighboring predicted sites. Next, to investigate if -248G/A could interfere with RNA folding, hence, affect the overall regulation process, we used Mfold [38] to simulate the RNA structures with either allele. The allele A lead to a strikingly different RNA structure that that of allele G (**Figure S5**). Altogether, data suggest that the c-248 G/A transition may affect the *EDN3* expression levels. Functional studies on this *EDN3* variant were not performed. Regulatory functional activity may vary from to cell to cell type and even if the *EDN3* variant were functional *in vitro*, we would never be able to ascertain its function *in vivo*, especially given the relevance of spatial-temporal regulation during developmental stages.

Importantly, Kenny and colleagues reported lower *EDN3* mRNA levels in ganglionic and aganglionic gut of HSCR patients devoid of CDS mutations compared to those in gut of non-HSCR individuals and suggested *EDN3* transcriptional deficit as a possible cause of aganglionosis [39].

Given that the *EDNRB* and *EDN3* variants only coexist in affected individuals (with both the EDNRB receptor and its ligand EDN3 compromised), it would appear that their joint effect is needed for the phenotype to manifest. This is in line with the essentiality of the interaction between EDN3 with ENDRB for the development of epidermal melanocytes and enteric neurons [40]. Thus, it is tempting to speculate that the transmission of the trait results from two independent mutations in two different genes of the same pathway. This family would exemplify a case of non-allelic non-complementation which occurs when recessive mutations in two different loci fail to complement one another and consequently, the double heterozygote exhibits a phenotype. Importantly, such mechanism has already been described in HSCR [17], [18].

Yet, while all individuals carrying both the *EDNRB* and the *EDN3* heterozygous mutations have virtually the same HSCR phenotype (isolated long segment), only one (II-5) member of the family has pigmentation problems. After an exhaustive literature search, we identified only three reports of HSCR patients with heterochromia iridum as the sole associated anomaly [41], [42]. No mutation screening had been done.

The *EDNRB* and *EDN3* mutations reported here have been submitted to the Leiden Open Variation Database (LOVD) v.3.0.

### CNV analysis

We also scanned the whole genome for rare CNVs. A total of 529 CNV regions and two chromosomal aberrations (CNV>1 Mb) were identified in this family. The chromosomal aberrations were transmitted by non-affected individuals to also non-affected individuals.

Of the 529 CNVs identified, 160 intersected with genes (genic-CNVs; 131 genes disrupted) and 369 CNVs were in non-coding regions of the genome. Among the genic-CNVs, 18 CNVs (35 genes disrupted) were present in at least one affected individual but also in unaffected family members. Likewise, among the non-genic CNVs, 62 were present in at least one affected individual but also in unaffected family members.

As a CNV reported in a healthy individual would be less likely to be pathogenic, all CNVs present in at least one affected individual were checked against the Database of Genomic Variants (DGV), a catalog of structural variations gathered from the analysis of 11,943 healthy individuals. All but 26 CNV regions (37 CNVs) and all chromosomal aberrations identified in this family had been also been detected among the DGV controls. None of the CNVs (including those no reported in DGV) were shared by the four affected individuals and carriers of the *EDNRNB* or *EDN3* mutations. Our data ruled out a direct role of CNVs in this familial disorder. Expectedly, no CNVs encompassing any of the WS4 candidate genes were detected.

Since II-5 is the only family member born with heterochromia iridum in addition to HSCR, we considered the possibility of the pigmentation problem being due to a genetic event independent of the *EDNRB/EDN3* mutations harboured by this individual.

We made use of the data provided by the CNV analysis to search for any specific feature that could account for the heterochromia iridum in that individual, bearing in mind that the search for another genetic lesion would require the scrutiny of the whole genome and that therefore, our CNV analysis is limited. Thus, we inspected the CNV profile of individual II-5 and paid attention to ***de novo*** **or inherited CNVs that were not present in any other family members (except for II-5 parents; I-1 and I-2) and had not been passed down to the next generation**. II-5 had 5 genes intersected by CNVs, 2 of which were *de novo* and 4 inherited (**Table** S2). Except for *ALDH2*, these genes were not only reported intersected in DGV but also in the Database of Chromosomal Imbalance and Phenotype in Humans Using Ensembl Resources (DECIPHER), which contains data on over 10,000 patients suffering from developmental disorders who harbor submicroscopic deletions or duplications. Interestingly, one of the ***de novo*** **CNV duplication (45.5 kb)** involved *DACH1* (dachshund homolog 1 (Drosophila); Attenuation of Forkhead signaling by t**he retinal determination factor**), a gene in chromosome 13q22 (6 Mb apart from ***EDNRB***) reported deleted in Decipher patient**s** affected with fullness of peri-orbital region or retinoblastoma. As for the non-genic CNVs present in II-5, we identified 3 inherited and 10 *de novo* CNVs (**Table S3**). Among the latter, a 38.5 kb duplication and a 20.9 kb deletion were detected in chromosome 13, 16.7 and 15.3 Mb upstream *DACH1* respectively. Importantly, iris heterochromia has been reported in a 13q-deletion syndrome patient without HSCR [43] as well as in infants with retinoblastoma. Whether these CNVs affecting *DACH1* or surroundings in chromosome 13q contribute to heterochromia iridum is unknown.

Overall, many of the CNVs reported in DGV are also reported in DECIPHER patients which indicate that the contribution of each CNV to the phenotype, if any, would be limited to the individual bearing them and this could account for the intra-familial variability observed, as exemplified by the phenotype presented by individual II-5. Of course, we cannot prove or disprove whether heterochromia iridum results from the CNVs unique to II-5 or/and from the *EDNRB/EDN3* mutations as reported in other patients with WS4. These CNVs may affect regulatory regions anywhere in the genome offsetting the synchronization and balance of the signaling network implicated in the disorder. Also, it should be born in mind that the heterochromia iridum phenotype could be due to a genetic mosaicism, that is, an independent event occurred during developmental stages, affecting only the embryonic cell lineage from which the eye pigmentation cells derive (in which case, we could not have detected the genetic lession).

In sum, our exhaustive genetic analysis shows that, in this Brazilian family of European ancestry, the manifestation of the HSCR phenotype may require mutations in two genes of the same pathway. The additional feature -iris heterochromia- presented by one affected individual could have resulted from an independent genetic event, such a *de novo* CNV affecting genes involved in eye development.

## Supporting Information

Figure S1 **Genomic structure and differential splicing of human** ***EDNRB*** **gene.** Black arrow indicates the ATG start site for isoform 3. Red arrow indicates the ATG start site for isoform 1 and 2 and is the location of the mutation found in the Brazilian family. SP: signal peptide.
(DOCX)

Figure S2 **The most likely** ***RET*** **haplotypes of the pedigree (A, C, G, T to 1, 2, 3, 4).** Individuals carrying the *EDNRB* mutation are indicated.
(DOCX)

Figure S3 **The most likely** ***EDN3*** **haplotypes of the pedigree were shown (A, C, G, T to 1, 2, 3, 4).** It showed that the c.-248G/A variation was transmitted from the I-1 and shared by all four affected individuals, II-1, II-3, II-5, and III-3 and one unaffected individual III-4.
(DOCX)

Figure S4 **Nucleic acid multiple sequence alignment of mammalian 5′UTR of** ***EDN3*** **.** The UCSC genome browser was used to identify sequence conservation features in the 5′UTR of *EDN3*. The red rectangle indicates the nucleotide c.-248G and it is highly conserved among species.
(DOCX)

Figure S5 **Predicted RNA secondary structures for the wild-type and mutated (-248G/A) 5′UTR of the** ***EDN3*** **.** The GGGGUGGU structure in the mutated 5′UTR of the *EDN3* formed a loop instead of a stem compared in the wild-type 5′UTR of the *EDN3*.
(DOCX)

File S1 **Supplementary material text.**
(DOCX)

Table S1 **TargetP 1.1 server prediction results.** Subcellular locations of different EDNRB isoform proteins were predicted by using TargetP 1.1 server. **SP**, a signal peptide; **mTP**, a mitochondrial targeting peptide; **M**, Mitochondrion; **S**, Secretory pathway; **RC**: Reliability class, from 1 to 5, where 1 indicates the strongest prediction.
(DOCX)

Table S2 ***Genes disrupted by CNVs in II-5.***
(DOCX)

Table S3 **Non-genic regions disrupted by CNVs in II-5.**
(DOCX)
