# Supplementary figures and images for: Genetic Analyses of a Three Generation Family Segregating Hirschsprung Disease and Iris Heterochromia
Source: PLoS One. 2013 Jun 26;8(6):e66631. doi: 10.1371/journal.pone.0066631 (PMC3694150; doi:10.1371/journal.pone.0066631)

**Figure S1:**


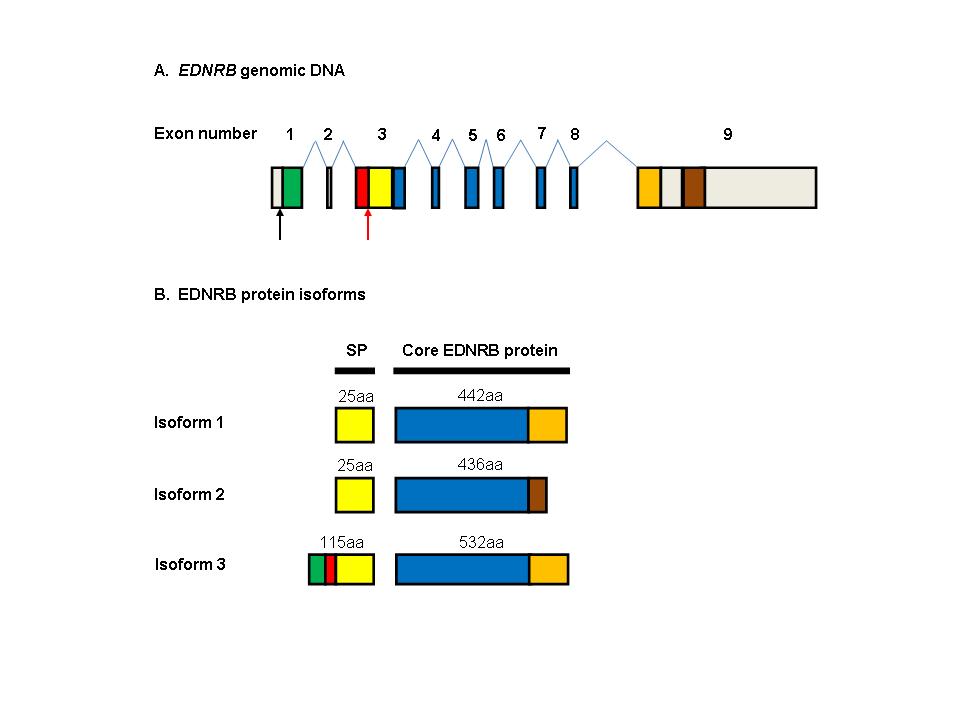

Supplement: Figure S1 — Genomic structure and differential splicing of human EDNRB gene. Black arrow indicates the ATG start site for isoform 3. Red arrow indicates the ATG start site for isoform 1 and 2 and is the location of the mutation found in the Brazilian family. SP: signal peptide. (DOCX) [file pone.0066631.s001.docx]

**Figure S2:**

**
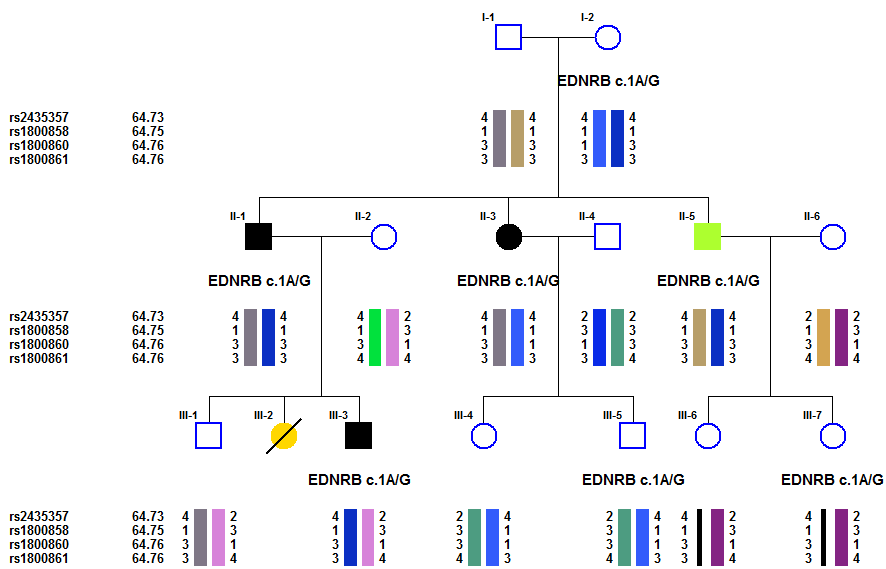
**

Supplement: Figure S2 — The most likely RET haplotypes of the pedigree (A, C, G, T to 1, 2, 3, 4). Individuals carrying the EDNRB mutation are indicated. (DOCX) [file pone.0066631.s002.docx]

**Figure S3:**

**
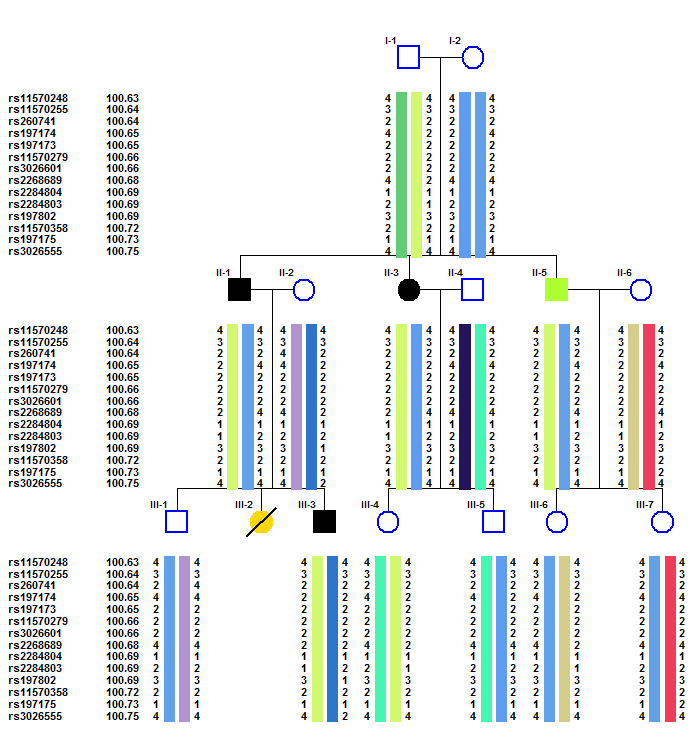
**

Supplement: Figure S3 — The most likely EDN3 haplotypes of the pedigree were shown (A, C, G, T to 1, 2, 3, 4). It showed that the c.-248G/A variation was transmitted from the I-1 and shared by all four affected individuals, II-1, II-3, II-5, and III-3 and one unaffected individual III-4. (DOCX) [file pone.0066631.s003.docx]

**Figure S4:**

**
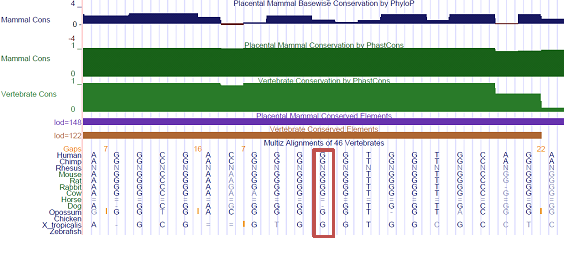
**

Supplement: Figure S4 — Nucleic acid multiple sequence alignment of mammalian 5′UTR of EDN3 . The UCSC genome browser was used to identify sequence conservation features in the 5′UTR of EDN3. The red rectangle indicates the nucleotide c.-248G and it is highly conserved among species. (DOCX) [file pone.0066631.s004.docx]

**Figure S5:**

**
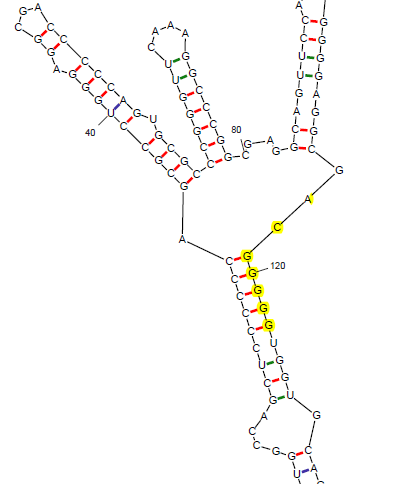

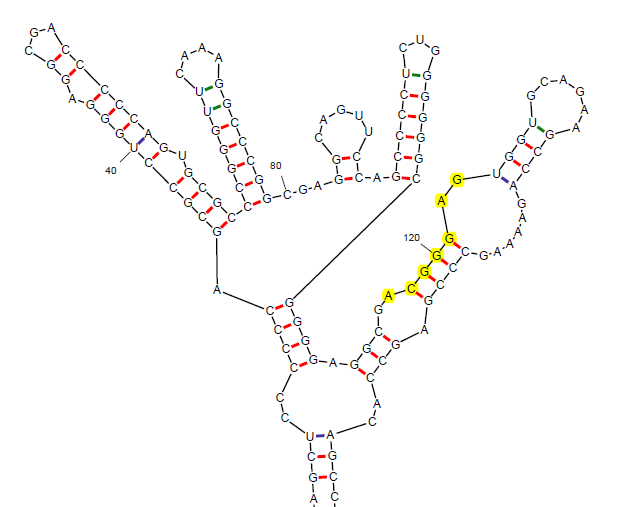
**

Supplement: Figure S5 — Predicted RNA secondary structures for the wild-type and mutated (-248G/A) 5′UTR of the EDN3 . The GGGGUGGU structure in the mutated 5′UTR of the EDN3 formed a loop instead of a stem compared in the wild-type 5′UTR of the EDN3. (DOCX) [file pone.0066631.s005.docx]
