# Supplementary material for: Genetic Analyses of a Three Generation Family Segregating Hirschsprung Disease and Iris Heterochromia
Source: PLoS One. 2013 Jun 26;8(6):e66631. doi: 10.1371/journal.pone.0066631 (PMC3694150; doi:10.1371/journal.pone.0066631)
Supplement: File S1 — Supplementary material text. (DOCX) [file pone.0066631.s006.docx]

# Supplementary Material

# Materials and Methods

## Bioinformatic analysis tools

PolyPhen-2: prediction of functional effects of human nsSNPs

http://genetics.bwh.harvard.edu/pph2/

UCSC Genome Browser

(<http://genome.ucsc.edu/cgi-bin/hgGateway>)

MITOPROT: Prediction of mitochondrial targeting sequences

(<http://ihg.gsf.de/ihg/mitoprot.html>)

TargetP 1.1 server

<http://www.cbs.dtu.dk/services/TargetP/>

## Copy number variations (CNVs)

### CNVs: quality control

CNV segments were predicted by two programs, PennCNV and QuantiSNP, the two most efficient CNV calling algorithms for Illumina data. To obtain high-confidence calls, we only used the overlapping region of CNVs called by PennCNV and QuantiSNP. Before selecting the overlapping CNV regions, quality controls were done separately for the CNV predicted by two programs.

For callings from PennCNV or QuantiSNP, CNVs shorter than 1kb or called with fewer than 3 probes were removed. In addition to these filtering criteria, we also remove CNVs with maximum Baye’s factor less than 10 for the predictions by QuantiSNP. In the analysis, only those regions intersected by CNVs called by both programs were included. All samples are of good quality for CNV calling as their genome-wide LRR standard deviation are all smaller than 3.5. CNVs might be artificially split by either of the calling programs. To circumvent this issue, adjacent CNVs of the same type (i.e. duplication or deletion) were merged if the length of gap in between was shorter than half of total length of the 2 CNV segments.

After quality control, a total of 529 CNVs in the pedigree remained for further analysis by using PLINK.

# References

1 Redon R, Ishikawa S, Fitch KR*, et al.* Global variation in copy number in the human genome. *Nature* 2006;444(7118):444-54.

2 Cooper GM, Coe BP, Girirajan S*, et al.* A copy number variation morbidity map of developmental delay. *Nat Genet* 2011;43(9):838-46.

3 Wang K, Li M, Hadley D*, et al.* PennCNV: an integrated hidden Markov model designed for high-resolution copy number variation detection in whole-genome SNP genotyping data. *Genome Res* 2007;17(11):1665-74.

4 Colella S, Yau C, Taylor JM*, et al.* QuantiSNP: an Objective Bayes Hidden-Markov Model to detect and accurately map copy number variation using SNP genotyping data. *Nucleic Acids Res* 2007;35(6):2013-25.

5 Dellinger AE, Saw SM, Goh LK*, et al.* Comparative analyses of seven algorithms for copy number variant identification from single nucleotide polymorphism arrays. *Nucleic Acids Res*;38(9):e105.
