# Supplementary material for: Genetic Analyses of a Three Generation Family Segregating Hirschsprung Disease and Iris Heterochromia
Source: PLoS One. 2013 Jun 26;8(6):e66631. doi: 10.1371/journal.pone.0066631 (PMC3694150; doi:10.1371/journal.pone.0066631)
Supplement: Table S1 — TargetP 1.1 server prediction results. Subcellular locations of different EDNRB isoform proteins were predicted by using TargetP 1.1 server. SP, a signal peptide; mTP, a mitochondrial targeting peptide; M, Mitochondrion; S, Secretory pathway; RC: Reliability class, from 1 to 5, where 1 indicates the strongest prediction. (DOCX) [file pone.0066631.s007.docx]

| Table S1: TargetP 1.1 server prediction results | | | | | |
| --- | --- | --- | --- | --- | --- |
| Name | Length (aa) | mTP | SP | RC | Localization |
| EDNRB isoform 1  EDNRB isoform 2  EDNRB isoform 3 | 442  436  532 | 0.057  0.057  0.817 | 0.945  0.945  0.035 | 1  1  2 | S  S  M |
| Subcellular locations of different EDNRB isoform proteins were predicted by using TargetP 1.1 server. **SP**, a signal peptide; **mTP**, a mitochondrial targeting peptide; **M**, Mitochondrion; **S**, Secretory pathway; **RC**: Reliability class, from 1 to 5, where 1 indicates the strongest prediction | | | | | |
