# Supplementary material for: Genetic Analyses of a Three Generation Family Segregating Hirschsprung Disease and Iris Heterochromia
Source: PLoS One. 2013 Jun 26;8(6):e66631. doi: 10.1371/journal.pone.0066631 (PMC3694150; doi:10.1371/journal.pone.0066631)
Supplement: Table S2 — Genes disrupted by CNVs in II-5. (DOCX) [file pone.0066631.s008.docx]

| **Table S2: Genes disrupted by CNVs in II-5.** | | | | | | |
| --- | --- | --- | --- | --- | --- | --- |
| **Genes** | **CNV regions** | | | **TYPE** | **Overlap with CNVs in DGV** | **Individuals** |
|  | **Chr** | **Start** | **End** |  |  |  |
| *NXPH1* | 7 | 8748207 | 8756695 | DEL | yes | II-5, I-2 |
| *ALDH2* | 12 | 110704079 | 110792590 | DUP | no | II-5, I-2 |
| *MAPKAPK5* | 12 | 110704079 | 110792590 | DUP | yes | II-5, I-2 |
| *DACH1* | 13 | 70979277 | 71024783 | DUP | yes | II-5* |
| *IGLL3* | 22 | 23993994 | 24076647 | DUP | yes | II-5, I-1 |
| *LRP5L* | 22 | 23993994 | 24076647 | DUP | yes | II-5* |
| **de novo* | | | | | | |
