# Supplementary material for: Genetic Analyses of a Three Generation Family Segregating Hirschsprung Disease and Iris Heterochromia
Source: PLoS One. 2013 Jun 26;8(6):e66631. doi: 10.1371/journal.pone.0066631 (PMC3694150; doi:10.1371/journal.pone.0066631)
Supplement: Table S3 — Non-genic regions disrupted by CNVs in II-5. (DOCX) [file pone.0066631.s009.docx]

| **Table S3: Non-genic regions disrupted by CNVs in II-5.** | | | | | |
| --- | --- | --- | --- | --- | --- |
| **CNV regions** | | | **TYPE** | **Overlap with CNVs in DGV** | **Individuals** |
| **Chr** | **Start** | **End** |  |  |  |
| 1 | 34878855 | 34883734 | DEL | yes | II-5, I-1, I-2 |
| 1 | 185983776 | 185988655 | DEL | yes | II-5* |
| 2 | 51912272 | 51919651 | DUP | yes | II-5* |
| 3 | 1757524 | 1762581 | DEL | yes | II-5* |
| 3 | 199338204 | 199375965 | DUP | yes | II-5* |
| 4 | 59662657 | 59672305 | DUP | yes | II-5, I-1, I-2 |
| 5 | 97074222 | 97121798 | DEL | yes | II-5, I-2 |
| 5 | 104749280 | 104763640 | DUP | yes | II-5* |
| 12 | 34566688 | 34569136 | DEL | yes | II-5* |
| 13 | 55287515 | 55325968 | DUP | no | II-5* |
| 13 | 56652161 | 56673101 | DEL | yes | II-5* |
| 22 | 24133932 | 24179589 | DUP | yes | II-5* |
| 22 | 24223281 | 24246179 | DUP | yes | II-5* |
| **de novo*. | | | | | |
